# Phenotypic plasticity and evolution of thermal tolerance in bacteria from temperate and hot spring environments

**DOI:** 10.7717/peerj.11734

**Published:** 2021-07-23

**Authors:** Enrique Hurtado-Bautista, Laura F. Pérez Sánchez, Africa Islas-Robles, Gustavo Santoyo, Gabriela Olmedo-Alvarez

**Affiliations:** 1 Departamento de Ingeniería Genética, Unidad Irapuato, de (Centro de Investigación y de Estudios Avanzados) del IPN, Irapuato, Guanajuato, México; 2 Instituto de Investigaciones Químico Biológicas, Universidad Michoacana de San Nicolás de Hidalgo, Morelia, Michoacán, México

**Keywords:** Phenotypic plasticity, Norms of reaction to temperature, Convergent evolution, Thermal tolerance, *Bacillus*, Hot spring, Temperature, Constrain, Life history, Sporulation

## Abstract

Phenotypic plasticity allows individuals to respond to the selective forces of a new environment, followed by adaptive evolution. We do not know to what extent phenotypic plasticity allows thermal tolerance evolution in bacteria at the border of their physiological limits. We analyzed growth and reaction norms to temperature of strains of two bacterial lineages, *Bacillus cereus sensu lato* and *Bacillus subtilis sensu lato*, that evolved in two contrasting environments, a temperate lagoon (T) and a hot spring (H). Our results showed that despite the co-occurrence of members of both lineages in the two contrasting environments, norms of reactions to temperature exhibited a similar pattern only in strains within the lineages, suggesting fixed phenotypic plasticity. Additionally, strains from the H environment showed only two to three degrees centigrade more heat tolerance than strains from the T environment. Their viability decreased at temperatures above their optimal for growth, particularly for the *B. cereus* lineage. However, sporulation occurred at all temperatures, consistent with the known cell population heterogeneity that allows the *Bacillus* to anticipate adversity. We suggest that these mesophilic strains survive in the hot-spring as spores and complete their life cycle of germination and growth during intermittent opportunities of moderate temperatures. The limited evolutionary changes towards an increase in heat tolerance in bacteria should alert us of the negative impact of climate change on all biological cycles in the planet, which at its most basic level depends on microorganisms.

## Introduction

Temperature is one of the most important physical factors that define a species fundamental niche (Hoffmann & Parsons, 1997). It affects many phenotypes, and numerous investigations on adaptation have focused on temperature to understand how it impacts physiological processes at the molecular level (Graumann & Marahiel, 1999; Bronikowski, Bennett & Lenski, 2001). Temperature affects a broad range of phenotypes, so it is used as a model to investigate how phenotypic plasticity evolves. Understanding phenotypic plasticity has become of high importance, given the expected temperature rise in the planet. Studies in ectotherm groups have suggested that variation in upper thermal limits is narrower compared to that of lower temperature and have suggested that evolution of heat tolerance is constrained. This asymmetry has been reviewed for terrestrial endo- and ectotherms, insects, amphibians and plants (Hoffmann, Chown & Clusella-Trullas, 2013; Araújo et al., 2013), and more recently an extensive data set was analyzed by Sunday et al. Sunday et al. (2019).

In contrast to the many studies that have been done in eukaryotes to determine their thermal phenotypic plasticity, in bacteria, there are few examples. Unlike the restrictions to the temperatures where eukaryotic organisms can thrive, Archaea and Bacteria can be found in extreme environments, from freezing (−40 °C) (Price & Sowers, 2004), to very hot (50 and to 100 °C) (Kristjansson, 1991). Their ubiquitous occurrence does not mean, however, that individual phyla or species have a broad spectrum of tolerance to temperature. Like eukaryotic ectotherms, individual bacterial taxa exhibit a limited temperature niche.

Phenotypic plasticity is the ability of an organism to exhibit distinct phenotypes when exposed to different environments (Murren et al., 2015; Van Gestel & Weissing, 2016), and allows organisms to acclimate to changes, extending the ecological range of a species, so they can survive exposure to pressures and creating the opportunity for assimilation (Waddington 1953, cited by Stearns (1989). Assimilation is the mechanism through which the initial plastic response allows diversification through genetic changes that stabilize the expression of the induced phenotype (Pigliucci & Murren, 2003). Interactions among phenotypic plasticity, life history and evolution persist for generations (Snell-Rood et al., 2018).

An organism genotype will define the delimiting range of environmental conditions to which it can acclimate. If an individual’s biological response to a changing environment is a function of gene content and its regulation, phylogenetically close organisms that experience similar environmental pressure may exhibit similar plasticity to respond to that particular stress, such as temperature. However, when species encounter changes in their environment, long term persistence will require the evolution of their plasticity. Since some habitats will, in fact, be less favourable to an organism survival and growth, costs and limits to the evolution of phenotypic plasticity are expected costs and limits to the evolution of phenotypic plasticity are expected (Snell-Rood et al., 2010).

**Figure 1:**
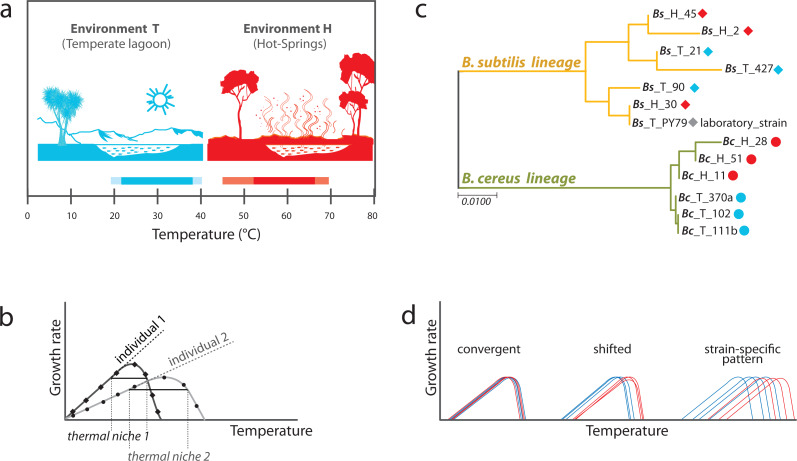
Experimental approach to analyzing phenotypic plasticity in two lineages of bacteria from contrasting environments. (A) Environments of isolation. Laguna Intermedia of Churince (Cuatrocienegas, México) (temperature range of 17 to 40 °C) and Hot-springs in Michoacán (Mexico) (temperature range between 48 and 70 °C). (B) Phylogenetic relationships for the *Bacillus* lineages under study representing mesophilic (M) and thermotolerant (T) life histories. *B. subtilis* and *B. cereus* from a Temperate-lagoon (blue), a Hot-spring (red) and a laboratory strain (PY79) (extensive phylogeny in Fig. S2). (C) A norm of reaction represents the performance of individuals under different environmental conditions. The entire curve is a trait, and the amplitude of the curve is the breath of the performance of the individual to a range of temperatures, and its thermal niche is the rank between the two *x*-values of 75% of maximal growth at optimal temperature (Bronikowski, Bennett & Lenski, 2001). (D) Scenarios of phenotypic plasticity between the lineages from two environments: individuals from different lineages or environments may exhibit the same response to temperature (convergence), a shifted reaction of the isolates from the hot-spring, or even strain-specific patterns of response to temperature regardless of genetic lineage (blue, environment T; red, environment H).

Temperature is a chosen variable in many studies that evaluate patterns of growth rate and survival in the population of bacteria. Growth rate represents a simple response variable of continuous phenotypes (Angilletta Jr & Angilletta, 2009). Phenotypic plasticity can be evaluated through reaction norms (Van Gestel & Weissing, 2016; Hendry, 2016 and Wolterek, 1909, cited by Stearns (1989)). Reaction norms are a description of how a phenotype varies as a continuous function depending on the environmental cues and is represented by a curve on a graph that plots a phenotype against an environmental factor (Fig. 1A). In a historical account on the study of norms of reaction, Stearns (1989) cites Dobzhansky’s writing: “what changes in evolution is the norm of reaction of the organism to the environment“. The complete reaction norm is a trait, and thus may be different between genotypes; it is genetically variable and can evolve and adjust.

Only 0.3% of the studies on plasticity-led evolution have been done in bacteria (Levis & Pfennig, 2020). The few studies on phenotypic plasticity to temperature in bacteria have been carried out on model laboratory bacteria, such as *Bacillus subtilis* (Zeigler & Nicholson, 2017) and *Escherichia coli* (see, for example, Bennett, Lenski & Mittler, 1992; Bennett & Lenski, 1993; Tenaillon et al., 2012). However, these strains may not be optimal to capture the complexity of phenotypic plasticity, as many traits may have been lost through passages under laboratory conditions. Genetic analyses have revealed genetic variation for thermotolerance under laboratory conditions (Mongold, Bennett & Lenski (1996) and references therein), but thermal phenotypic plasticity of bacteria resulting from selection pressures in nature remains largely unknown.

The *Bacillus* genus is characterized by endospore-forming bacteria, and representatives of this genus are present in almost every wild environment around the world (Ravel & Fraser, 2005; Hernández-González, Moreno-Hagelsieb & Olmedo-Álvarez, 2018; Souza et al., 2018). *Bacillus* is an interesting model to study phenotypic plasticity. Its ability to develop a highly resistant spore allows survival at a temperature that would be lethal for the vegetative cell, thus allowing it to survive extreme changes. How then could refinement of its phenotype to tolerate higher temperatures occur if the immediate response of these bacteria to stress is sporulation? The fact that the *Bacillus* can tolerate heat in their sporulated form does not make the *Bacillus* species termpphiles, as most species of this genus cannot grow above 50 °C. Some *Bacillus* species have been recovered from extreme environments and are thermophiles, such as *Bacillus infernus* and *Bacillus fumarolis* (Logan & Allan, 2008), but the best-studied thermophilic genus in the Firmicutes family are usually classified as different genera, such as *Geobacillus, Thermaerobacter* and *Thermobacillus* (Fritze, 2004).

Hot springs have been recurrent systems for investigating in natural systems the capability of a given species to occupy different thermal niches. Weltzer and Miller showed that *Chloroflexus* strains from the White creek thermal gradient have diverged in their growth temperature range for growth (Weltzer & Miller, 2013). On the other hand, laboratory strains of the *Synechococcus* A/B group of cyanobacteria isolated from different temperatures sources (Yellowstone and Oregon hot springs) are ecological specialists with divergent temperature ranges for growth (Klatt et al., 2011).

Although some *Bacillus* strains that represent mesophilic clades are sometimes isolated from hot-springs, there is typically little information of their taxonomy and even of their temperature tolerance. It is possible that, with a few exceptions, many strains in the *Bacillus* genus isolated from hot-springs are not thermophilic and they tolerate heat as spores. For instance, seldom are mesophilic *Bacillus* species, such as *Bacillus cereus* and *B. subtilis*, recovered from hot springs. Being so ubiquitous, can they extend their range of temperature tolerance and evolve into thermophilic strains?

Among the numerous *Bacillus* species recognized, two mesophilic lineages have been extensively studied, the *B. cereus sensu lato*, that includes *B. cereus, Bacillus thuringiensis*, and *Bacillus anthracis* (Bazinet, 2017 ), and the *B. subtilis* complex, that includes *B. subtilis, Bacillus amyloliquefaciens, Bacillus licheniformis*, and *Bacillus pumilus*, among others (Fritze, 2004; Zeigler, 2011). The most recent study by Hernández-González, Moreno-Hagelsieb & Olmedo-Álvarez (2018) showed that the *B. cereus* and *B. subtilis* lineages form two distinct clades. Additionally, the *B. cereus* strains typically possesses a genome of between 5 to 6 mega base pairs, while the genome of *B. subtilis sensu lato* is around 4 mega bases long (Alcaraz et al., 2010).

Bacteria, in general, exhibit considerable genetic variability, in part from their ability to interchange genes through horizontal gene transfer. Within the genus *Bacillus* there is a large intraspecies phenotypic variability (Rodríguez-Torres et al., 2017) and a significant variation in the genetic repertoire through microevolution (Gómez-Lunar et al., 2016). Up to 30% of genes may be only partially shared within a bacterial species (Tettelin et al., 2005). Organisms that have evolved in a given environment may be constrained in their response, maybe from having adjusted their genes to their particular environment (Hernández-González, Moreno-Hagelsieb & Olmedo-Álvarez, 2018). If this was the case, their life history could come very close to constitute its genetic history as well. Bacteria are excellent models to explore the evolution of phenotypic plasticity, through the evaluation of reaction norms to temperature. Their genetic variability makes them special cases to explore whether their genetic architecture is so malleable that their norms of reaction change to adjust to the environment or if, on the contrary, despite this variability, their reaction norm is fixed, such that the norm of reaction can be a trait of the phylogeny. At the molecular level, temperature response has been extensively studied in bacteria and particularly the response elicited by both cold- and heat shock (Barria, Malecki & Arraiano, 2013; Richter, Haslbeck & Buchner, 2010). We do not know, however, whether the large repertoire of genes required for thermal adaptation constrains the evolution of tolerance.

In this work, we evaluated phenotypic plasticity through reaction norms to thermal tolerance in a lineage Vs. environment model in bacteria from natural settings. By examining the evolution of upper thermal limits in bacterial strains from contrasting environments, it is possible to evaluate trait limits related to evolutionary history. The bacterial strains used in this study comprised two lineages within a genus, *B. cereus sensu lato* and *B. subtilis sensu lato*. The strains were obtained from a hot-spring (environment H) and a temperate lagoon (environment T), both in Mexico, and were used to address the following questions: Do individuals of closely related lineages with a similar history of temperature selection (either in the hot-spring or in the temperate lagoon) exhibit convergence in their norms of reaction? Do the *Bacillus* from the hot-springs evolve tolerance to temperature in their vegetative stage?

Our results showed that reaction norms to temperature of the different individuals reflected their evolutionary history. The *B. cereus* and *B. subtilis* lineages each exhibited distinct response patterns, suggesting that the genetic architecture of each lineage constrained their phenotypic plasticity despite their sharing of environmental conditions. For both lineages, covariation was observed between environmental temperature and thermal tolerance phenotype, suggesting temperature adaptation. The individuals from the hot-springs were, as expected, more tolerant to hot temperature, yet, their tolerance did not match the hot-springs temperature suggesting, particularly for the *B. cereus* lineage, that its ecological strategy depends mainly on sporulation. These results may suggest that sporulation decreases the opportunity for evolving tolerance and that the lineage in its vegetative state is already close to its thermal tolerance limit.

## Materials and Methods

### Evaluation of mesophile and thermophile strains

**Figure 2:**
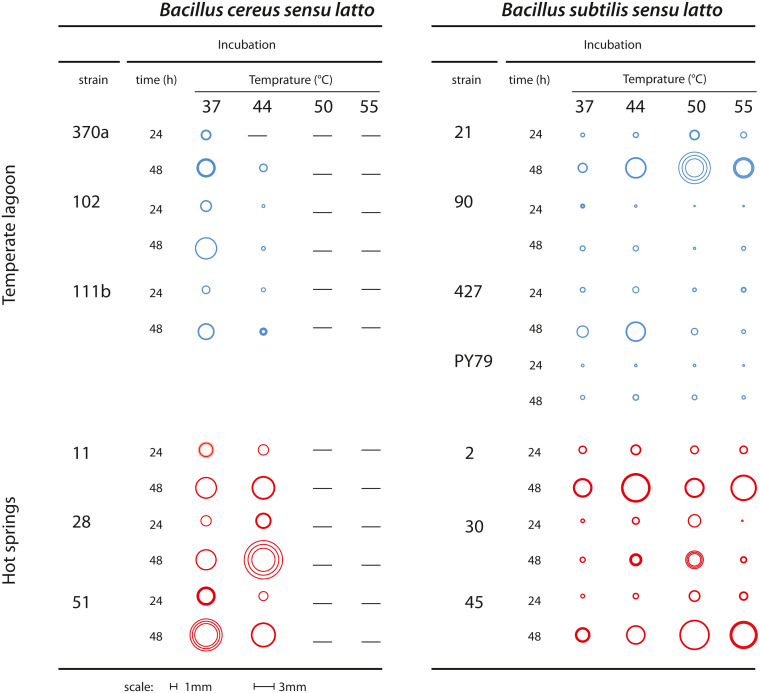
Growth of colonies of *Bacillus.* strains from the *cereus* and *subtilis* lineages and environments at different temperatures. Graphical representation of the size of single colonies after 24 and 48 h incubation at different temperatures (37, 44, 50 and 55 °C) on semisolid Marine Medium. Three isolated colonies were chosen, and their size represented in a circle; the inner circles and the outer circles represent, respectively, the lower and upper limits of the standard deviations error bars. Dashes indicate that no growth was observed. Figure S1 shows photographs from some of the plates.

Bacteria classified as mesophilic can tolerate a range of 18 to 45 °C, while thermotolerant bacteria tolerate from 22 to 60 °C. Both mesophilic and thermotolerant bacteria have growth optima below 50 °C. Thermophilic bacteria, in contrast, have an optimal growth temperature above 60 °C (Canganella & Wiegel, 2011). Mesophilic *Bacillus* were collected from the Churince water system, where daily and seasonal variations in temperature have been recorded. The spring is fed by subterranean water and the temperature in this system range from 18 to 31 °C (Cerritos et al., 2011). The thermotolerant *Bacillus* strains in this study were collected in the geothermal system of the Araro region, located in the central part of Mexico, inside the trans-Mexican volcanic belt located in the Michoacan state. The samples were directly collected from microbial mats of a hot spring at a depth of 30–50 cm from the surface. A collection of bacteria was obtained through direct plating on different media. Since there was no treatment at high temperature to select for spores (Pérez-Gutiérrez et al., 2013), we cannot know if the recovered strains were in a vegetative or sporulated form. The dominant bacteria in this extreme environment were firmicutes, inhabiting the microbial mats in the springs (Prieto-Barajas et al., 2017). Temperature and physicochemical parameters were evaluated in different seasons and found to fluctuate between 45 and 55 °C (Bonita spring) and 63 o 74 °C (Tina hot-spring) (Prieto-Barajas et al., 2017). For this study, we chose sets of strains from two closely related taxa, both of the *Bacillus* genus (as explained below). Six strains were isolated from the Temperate intermediate lagoon (environment T) and six more from the hot-spring in Michoacan (environment H) (Fig. 1A). We included a *B. subtilis* laboratory strain, PY79, which is presumably mesophilic (Youngman, Perkins & Losick, 1984). Bacterial strains were kept in frozen stocks at −70 °C. To observe the phenotype of their colonies selected strains were streaked out on semisolid Marine medium and incubated for 24 h to 48 h at 37, 44, 50 and 55 °C. To ensure that neighbouring colonies were not limiting the colony size, we also plated dilutions and incubated the plates at the stated temperatures. We took pictures from plates with at most 20 colonies and chose for each strain three well-isolated colonies to measure their diameter. Figure 2 shows a diagram of colonies from each strain at the different temperatures photographs of colonies at different temperatures are shown in Fig. S1 and colony diameter data is shown in Table S1.

### Strain selection from 16S rRNA phylogenetic reconstruction analysis

PCR of 16S rRNA genes was performed on DNA from a collection of strains from the temperate lagoon and the hot-springs. Forward and reverse sequences were obtained by Sanger dideoxy sequencing, and were edited by cutting off low-quality segments and concatenated as a consensus sequence for each gene using Bioedit version 7.0.5.3. After an alignment using Muscle,a phylogenetic reconstruction was done with Mega 7.0.26 (see Fig. S2). We chose for this study six strains that grouped with the *B. cereus sensu lato* (were 99% similar based on sequence variation of the 16S rRNA gene, accession number: Bc_370a MK850162.1; Bc_102 MK880370; Bc_111b MK850167.1; Bc_28 MK850171.1; Bc_11 MK850156.1; Bc_51 MK850144.1) and six strains from the *B. subtilis* lineage (accession number: Bs_21 MK850173.1; Bs_90 MK850153.1; Bs_427 MK850159.1; PY79 MK850170.1; Bs_2 MN180832; Bs_30 MK850157.1: Bs_45 MK850154.1). For the simplified phylogeny shown in Fig. 1B, a gene alignment of the 16S rRNA gene of the 13 chosen strains was carried out using Muscle and tree construction was performed by the Maximum Likelihood method with the HKY+G substitution model using MEGA version 7.0.26 (Hall, 2013).

### Determination of growth rates evaluation and norms of reaction

Bacterial isolates from −70 °C stocks were reactivated on semisolid Marine Medium (Pérez-Gutiérrez et al., 2013) at 37 °C for 20 hrs. One colony from each strain was inoculated into to 50 ml Falcon tubes with 5 ml of Marine medium and incubated overnight at 37 °C in a shaking incubator. A 50 µl aliquot was transferred to tubes with fresh medium and and growth was monitored until exponential growth was reached. Five µl of each culture was then used to inoculate 200-well microtiter plates (Bioscreen C, Labsystems, Helsinki, Finland) previously filled with 175 µl of Marine medium-broth. Growth measurements were carried out with a 420–580 nm filter, with three replicates and optical density was measured every 30 min for 20 h (Table S2). For reaction norms to temperature growth curves were obtained at 17, 27, 37, 41, 43, 46, 49 and 55 °C. Doubling time was calculated using an exponential model for growth (Table S3). Differences in the reaction norms among groups were evaluated with a t-Test (Table S4). Multiple statistical Anovas (0.05 of significance) for comparison throughout all the entire reaction norms were performed in Statgraphics version 15.2.06. The optimal temperature was defined as that with the maximum peak in growth rate throughout the range of tested temperatures. The thermal niche was calculated as the range of temperatures over which the observed doubling rate equaled or exceeded 75% maximum doubling time (Bronikowski, Bennett & Lenski, 2001) (see Fig. 1). To compare species, we grouped data of doubling time at each temperature of *B. subtilis* strains from both environments and compared against the grouped *B. cereus* data. To compare by environment, we grouped the growth rate data of each temperature of *B. subtilis* plus *B. cereus* from each one of the places of origin. An ANOVA in R package 3.6.2 (with significance level at 0.05) to identify statistical differences in the double comparison.

### Evaluation of heat tolerance and sporulation at different temperatures

Bacterial isolates from −70 °C stocks were reactivated on semisolid Marine Medium (Pérez-Gutiérrez et al., 2013) at 37 °C for 20 hrs. One colony from each strain was inoculated into 50 ml Falcon tubes with 5 ml of Marine medium and incubated overnight at 37 °C in a shaking incubator. Cultures were started in Marine medium using nephelometric flasks and grown to exponential phase, monitoring growth in a Kett Summerson colorimeter (red filter). The samples were divided into three tubes that were incubated at 37, 44 or 50 °C. The cultures were maintained overnight with shaking. Dilutions were made and plated on Marine agar to obtain the total viable count (both vegetative cells and germinating spores). Spores counts were obtained by treating an aliquot of the cultures at 80 °C for 30 min before plating. The plates were incubated overnight at 37 °C to obtain colony-forming units (Table S5).

## Results

### Evaluation of phenotypic plasticity in a *Bacillus* two-lineages model, each with members that evolved in contrasting temperature environments

We studied *Bacillus* isolates in a classical gene ×environment setup, using strains isolated from sediment in the Intermediate lagoon of Churince in Cuatrocienegas, Coahuila (Pérez-Gutiérrez et al., 2013) and isolates cultivated from the mats of hot-springs in Michoacan (Prieto-Barajas et al., 2017). The two different environments appear to have non-overlapping temperature ranges. The temperate water and sediment of the Churince system, from which part of our microbial collection was obtained, has a temperature that fluctuates between 18 to 36 °C (we refer to this as Environment T), while that of the hot spring fluctuates between 45 and 70 °C (Environment H) (Fig. 1A). *Bacillus* strains are easily recovered from both the T and H environments. The strains used in this work have been previously reported (Prieto-Barajas et al., 2017; Pérez-Gutiérrez et al., 2013). Phylogenies based on 16S rRNA gene of several strains from the different environments were obtained to select those that would be genetically closest (Fig. S2). Emphasis was made in clades *B. subtilis sensu lato* and *B. cereus sensu lato*. These lineages are referred to as Bc and Bs, for short. We chose three strains from each *Bacillus* lineage and from each environment (H and T) to evaluate phenotypic plasticity through comparative norms of reaction. We also included in the study a laboratory strain of *B. subtilis*, strain PY79 (Youngman, Perkins & Losick, 1984). A simplified phylogeny is shown in Fig. 1B.

### Growth and colony size differences between the Bc and Bs lineages challenged at high temperature

Colony growth was evaluated on semisolid marine medium with incubation at different times and temperatures (Fig. 2 and Fig. S1). We observed growth and colony size differences between strains from the Bs and Bc lineages. Regardless of the environment of isolation, the Bs lineage strains were more tolerant to high temperature than those of the Bc lineage, although the size of single colonies was generally smaller than those of the Bc lineage. The strains from the Bs lineage from the environment T could still grow at 50 °C, although forming small colonies. In two of these strains Bs-T-427 and the laboratory strain PY79, some growth was observed even at 55 °C. In contrast, none of the strains from the Bc lineage could grow at 50 °C. The strains from the Bc lineage from environment H exhibited perceptibly larger colonies than their counterparts from environment T, and even at 44 °C grew robustly, suggesting adaptation of these strains to fast growth at 44 °C. Noteworthy, even when challenged at higher temperature (44 °C), colony growth was sustained, since colony size after 48 h incubation was noticeably larger than at 24 h incubation (see Fig. 2 for a schematic of colony size).

### Distinct norms of reaction to temperature of the two Bacillus lineages that co-occur in the Churince temperate lagoon

Phenotypic plasticity was assayed through the norm of reaction to temperature for each strain. Growth curves for each strain were obtained at temperatures from 17 to 55 °C. The thermal niche of each strain was calculated as the range of temperatures over which the observed doubling time equaled or exceeded 75% of the peak doubling time (Bronikowski, Bennett & Lenski, 2001) (Fig. 1C). In this gene ×environment evaluation different norm of reaction scenarios were possible (Figs. 1C and 1D): In one scenario (fixed plasticity), bacteria from both lineages could exhibit the same response to temperature, regardless of the environment where they had evolved. In a second scenario, a shift of tolerance towards higher temperature in both lineages would be observed. In this last case, the selective environmental pressure would result in a convergent phenotypic response regardless of the lineage. In this scenario, strains from the Bs and Bc lineage would exhibit the same response to temperature within each environment. In a third scenario, even if strains from environment H tolerated higher temperature than those from environment T, each individual would exhibit dissimilar norms of reaction to temperature, regardless of the lineage.

**Figure 3:**
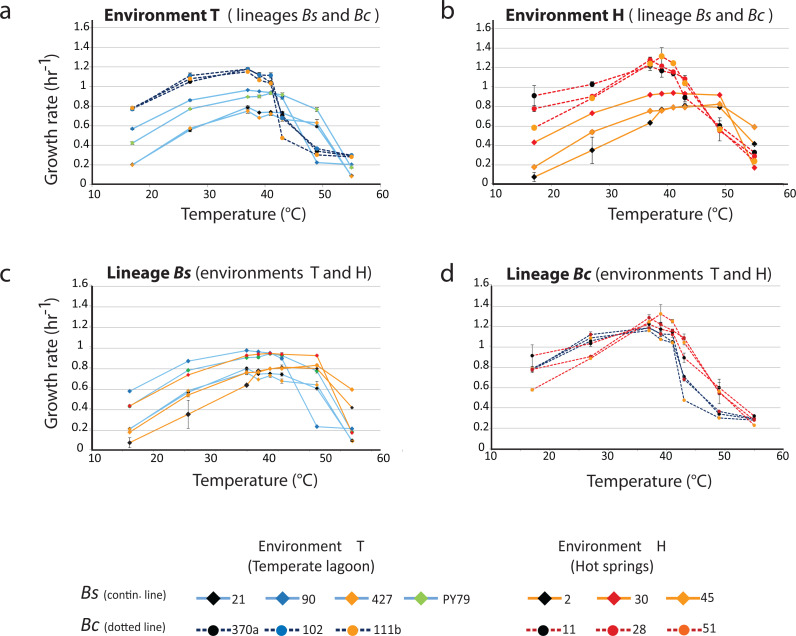
Lineage-specific phenotypic plasticity as a response to temperature. (A) Norms of reaction to temperature of the *B. cereus* and *B. subtilis* lineages from the Temperate lagoon. (B) Norms of reaction to temperature of the *B. cereus* and *B. subtilis* lineages from the hot-springs. (C and D) Combined data from curves in A and B, to highlight similarities in the response within the *B. subtilis* (C) and *B. cereus* lineage (D). Dashed lines, *B. cereus* lineage, continuous lines, *B. subtilis* lineage. Dark and light blue, Temperate lagoon strains. Red and orange, Hot-springs strains. Response was evaluated at temperatures 17, 27, 37, 43, 46, 49, 55 °C.

Figure 3A shows the profiles of the norms of reaction to temperature for the strains from the temperate lagoon (T environment). It is observed that despite sharing the same environment, the norms of reaction of the strains from the two lineages did not converge. The strains from the T environment had norms of reaction with a distinct pattern, clearly different between lineages. All Bc strains exhibited a higher growth rate at temperatures from 17 to 40 °C, but growth fell sharply above this temperature. The strains from the Bs lineage exhibited a lower growth rate at all temperatures but could still sustain growth 2 °C above the Bc strains. Despite experiencing the same fluctuations in temperature in the sediment of the small Churince lagoon, the two lineages could be easily discerned by their norm of reaction, suggesting differences in phenotypic plasticity.

### Norms of reaction to temperature, a trait that differentiates the *Bacillus* lineages that co-occur in the hot-spring

Figure 3B shows the profiles of the norms of reaction to temperature for the strains from the hot-spring. Norms of reaction to temperature of strains were more similar within lineages. A stronger selective pressure to adapt to increasing temperature in the hot-spring did not lead to convergence of the two lineages in response to temperature; it seems thus that the distinct lineage-specific norm of reaction to temperature is a “stable” trait. As observed for these lineage strains from the lagoon, a higher growth rate of the Bc lineage strains, followed by an abrupt drop was observed compared to that of the Bs strains, for which their growth pattern stretched smoothly towards higher temperatures.

### Evolution of tolerance to higher temperature of strains from the hot-spring

The strains of the Bs lineage in the H environment exhibited a wider range of temperature tolerance than those of the Bc lineage. The strains of Bs lineage sustained growth rate at higher temperature, and they reached a plateau and maintained the same growth response for a wide range of temperatures, to the point that no single optimal growth temperature could be defined. Growth only dropped at temperatures close to 50 °C (Fig. 3C).

In contrast, the strains from the Bc lineage from the hot-spring had a higher growth rate than those of the strains from the Bs lineage through all the temperatures tested, until the temperature reached 42 °C, and then an abrupt drop in growth ensued. This can be clearly observed when comparing Fig. 3C, for lineage Bs, with Fig. 3D, for lineage Bc, as these graphs combine the norms of reaction from the T and H environments. Clearly, both Bs and Bc strains from the H environment exhibited higher tolerance for growth at temperatures above 42 °C, than those from environment T, suggesting that the strains have adapted to grow at a higher temperature. However, tolerance to temperature does not exceed more than a couple of °C above the maximal temperature exhibited by the strains from the T environment. Even though the hot springs measured temperature fluctuate from 46 to 70 °C, these lineages did not grow at temperatures above 45 °C. Noticeably, there was a tendency for strains from environment H to grow less at temperatures below 37 °C. An increased higher temperature combined with a decreased tolerance at lower temperature suggests a trade-off between phenotypes that limits phenotypic plasticity. The fact that each lineage exhibited a particular pattern and none of the strains in the Bc lineage could grow beyond 45 °C, supports the concept that the degree to which an organism can alter its phenotype is governed by its genetic architecture, that delimits the range of environmental conditions to which it can adapt and where evolution may not act beyond, for this specific selective pressure.

### Thermal niche of strains from hot-springs and from the temperate lagoon

**Figure 4:**
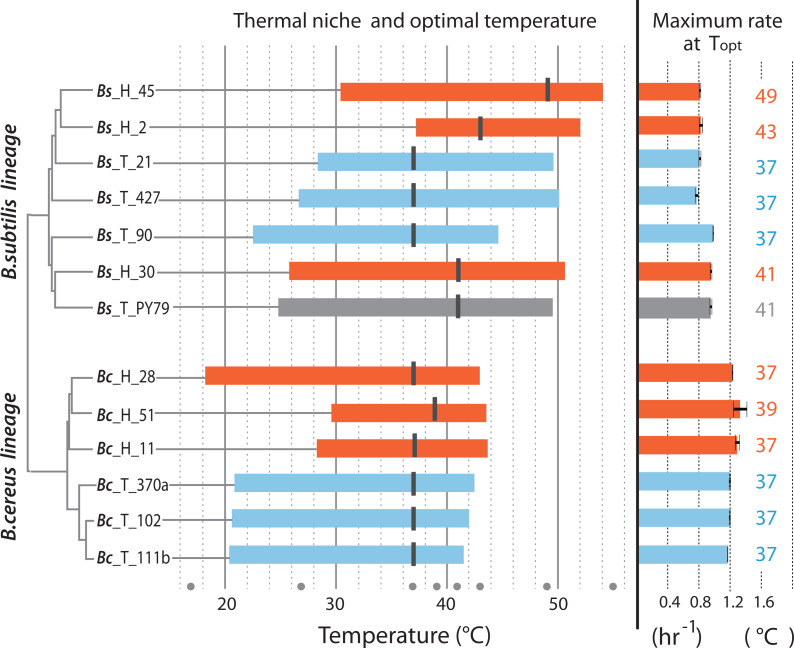
Thermal niche of the *B. cereus* and *B. subtilis* lineages from the temperate lagoon and the hot-springs. Thermal niche is simplified as the range between the two *x*-values of 75% of maximal growth at optimal temperature as described by Bronikowski, Bennett & Lenski (2001) and depicted in Fig. 1B. Rectangles depict range at or above 75% of maximum growth of strains from the Temperate lagoon (blue) and the Hot-springs (red). Black bars across the rectangle indicate the optimal temperature for growth (strain Bs H 30 exhibits maximum growth at two temperatures). Gray dots, temperatures of evaluation 17, 27, 37, 43, 46, 49, 55 °C. Maximum growth rate at optimal temperature for each strain is depicted to the right.

Figure 4 is a summary of the measured parameters for both mesophilic and temperature-tolerant strains in both lineages, including thermal niche, optimum temperature and specific growth reached by individual strains at their optimal temperature (37 °C for most strains). The amplitude of the curve in the norm of reaction is the ability of each individual to grow at a range of temperatures, while its thermal niche is the rank between the lower and upper values of 75% of maximal growth at an optimal temperature, as described by Bronikowski, Bennett & Lenski (2001). Regardless of lineage, all strains from the hot-spring exhibited a shift in their capability for growth at a higher temperature. For two strains in the Bc lineage from the H environment (strains Bc-H-51 and Bc-H-11), the extension in the capacity for growth at higher temperature seemed to impose a trade-off for growth at the lower temperature, and only strain Bc-H-28 exhibited increased tolerance without trade-off at low temperature. One of the strains from the Bs lineage from environment H (Bs-H-2) also exhibited a markedly lower capacity for growth at a lower temperature. All other strains from environment H exhibited a similar capacity for growth at a lower temperature as those from environment T, suggesting that they possessed the ability to grow at a wider range of temperatures.

The optimal temperature for growth for all strains from both the Bc and Bs lineages that evolved in the T environment was 37 °C. Notably, all strains in the Bs clade from the environment H have a shifted optimal growth temperature to 43 °C (Bs-H-30 and Bs-H-2) and even to 49 °C (Bs-H-45). Interestingly, Bs-H-30 exhibited a plateau of optimal growth with a second optimal peak at 49 °C. It is intriguing that the laboratory strain, Bs PY79, exhibited a wide range of growth and even an optimal growth at 41 °C. The greater tolerance to temperature of *B. subtilis* compared to *B. cereus* lineage agrees with data obtained on semi-solid medium (Fig. 2 and Fig. S1).

It is evident that the strains from the Bc lineage from the H environment exhibited only 1 to 2 °C advantage in temperature tolerance and increased minimally their maximum capacity for growth. However, the specific growth rate of the Bc lineage strains, from either environment was always higher than that of strains from the Bs lineage, and this growth ability seems to be a trait of the species (Fig. 4). This was also observed in the formation of larger colonies on plates (Fig. 2 and Fig. S1).

**Figure 5:**
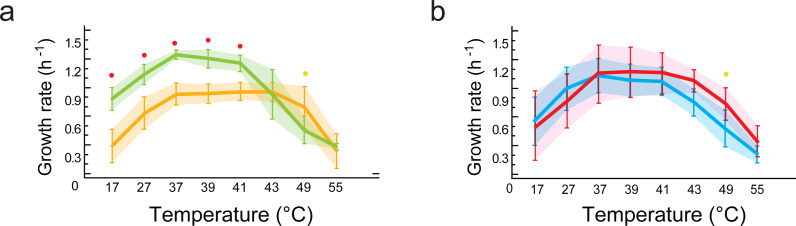
Phenotypic plasticity is constrained by genetic lineage despite evolution in different environments. (A) Statistical analysis of growth rates with data grouped by lineages (*B. subtilis* in yellow vs.*B. cereus* in green). Significant differences observed for growth response at five temperatures (red dots at 17, 27, 37, 39, 41 °C). (B) Statistical analysis of growth rates with data grouped by environment (environment T in blue vs. environment H in red). No statistically significant differences in growth were observed when comparing data grouped by environment (T versus H). Shaded areas show overlap in all temperatures. Each curve represents the mean of growth rates allocated to each set of strains. An ANOVA in R package 3.6.2 (at significance level at 0.05) was used to identify statistical differences in the double comparison, and intervals of confidence are shown with shaded area.

Notably, within the Bc lineage, both optimal growth temperature and maximum optical density reached by the different strains measured at 37 °C, was similar to that of their counterparts from the temperate lagoon. The growth dynamics of Bc lineage isolates from the H environment did not exhibit the phenotypic plasticity expected for an organism from an environment that appears to always be above 46 °C. A graphic of the grouped data of the T and H strains from each of the lineages shows a clear lineage-specific norm of reaction to temperature (Fig. 5A), with the only point of convergence at 43 °C. The Bs lineage appeared to have higher phenotypic plasticity to adapt its growth to a wider range of temperatures. The Bs lineage strains exhibit not a single optimal temperature, but an ample range of temperatures at which they sustain growth, possibly as a result of physiological plasticity. The strains within each lineage conserved a characteristic pattern in the norm of reaction that did not converge in their shared environments (Fig. 5A). No statistical differences were observed when strains from T and from H were combined (Fig. 5B) to compare environments, suggesting that species lineage exhibited a stronger signal in plasticity than the environment.

**Figure 6:**
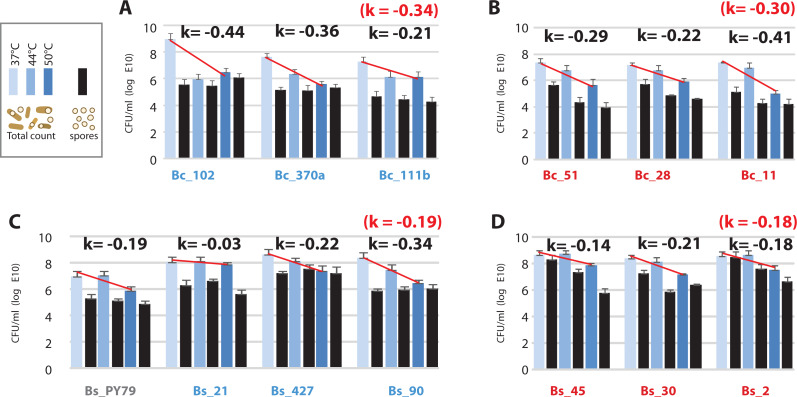
Viability of the *Bacillus* from the temperate lagoon (environment T) and hot-spring (environment H) environments at temperatures above their optimal temperature for growth (37 °C). (A) Bc strains from environment T; (B) Bc strains from environment H; (C) Bs strains from environment T; (D) Bs strains from environment H. Log phase cultures (37 °C) were transferred to shaking incubators at 37, 44 and 50 °C for 24 h. Total CFU/ml was obtained by plating on Marine medium (light-, medium- , and dark-blue bars, are CFU/ml from cultures incubated at 37, 44 and 50 °C, respectively) and spores counts (black bars) were obtained by plating the same cultures after a 30 min heat treatment at 80 °C. K represents the mean of sensitivity to temperature for each group, calculated as k = ln (N2-N1)/(T2-T1) (Table S5) and represented by a straight line in semilogaritmic plot.

### Asmesophilic bacteria, *Bacillus* strains lose viability at temperatures above their optimumbut sporulateat all temperatures

The observed increased tolerance of strains from the hot-spring by only two °C is incompatible with the tolerance required for survival in a hot-spring, above 46 °C. We measured viability of cells after incubation at 37, 44 and 50 °C for 24 h, as colony forming units (CFU) and also quantitated spores in these cultures. Our results showed that viability decreased at 50 °C in all strains, but the loss of viability was higher for the Bc lineage (*k* =  − 30) than for the Bs lineage (*k* =  − 19) (Fig. 6). Consistent with the norms of reaction, strains from the Bc lineage from the hot-spring were more tolerant at 44 °C than their counterparts from the temperate lagoon (Fig. 6). All strains sporulated at all temperatures, though the per cent sporulation was variable among strains and even among experimental replicates, consistent with the heterogeneity observed for *B. subtilis* populations (Dubnau & Losick, 2006; deJong, Veening & Kuipers, 2010) (Table S5).

**Figure 7:**
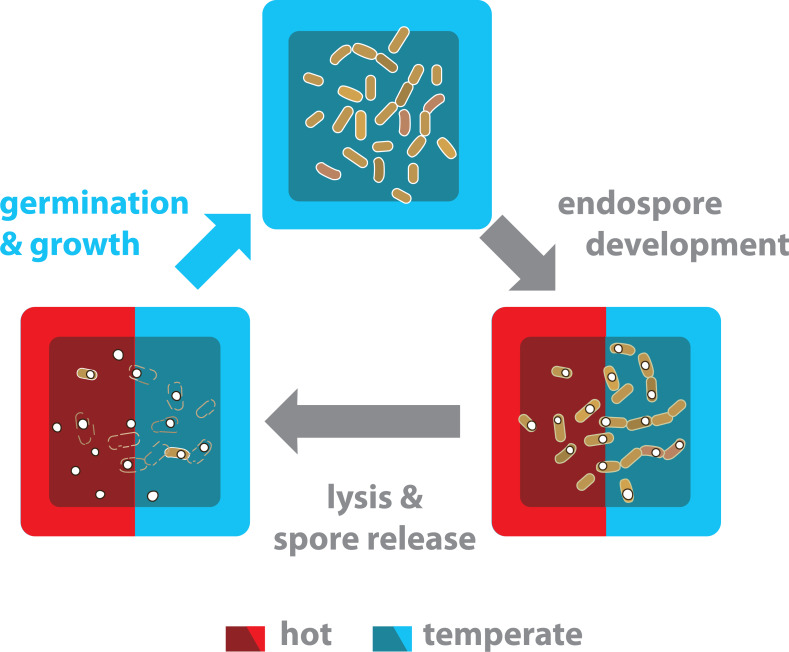
The life cycle of endospore-forming *Bacillus* from both the hot-spring and temperate environments is completed in a temperate environment. Life cycle of bacteria from the *Bacillus* genus has three different physiological stages: vegetative growth, sporulation and germination. Nutritional and environmental stress may trigger a developmental program to produce endospores. Released mature spores are highly resistant and can survive for years. Significant sporulation is observed in all strains and culture conditions (Fig. 6). The *Bacillus* strains from the hot-spring belong to mesophilic clades (*subtilis* and *cereus*) and although they have evolved more tolerance to temperature, it is not enough to survive in a vegetative state in the hot-spring temperature. Thus, under the hot-spring conditions, these *Bacillus* lineages most probably perdure as spores and only under favourable temperature conditions do they germinate and resume growth.

Our results suggest that the strains from the hot-spring most likely survive as spores within the hot-spring, completing their life cycle of germination and growth only at moderate temperatures (Fig. 7). This scenario may occur periodically, possibly due to water outflow from the hot-spring, during the rainy season. Our results suggest that the *Bacillus* from the hot-spring have evolved limited temperature tolerance in their vegetative phase as their life cycle allows that a fraction of their population enters sporulation and tolerate the high temperature as spores.

## Discussion

It has been observed that realized niches for species in warm environments are closer to their physiological limits (Araújo et al., 2013). Which is the physiologicallimit for a sporulating bacteria, and will they evolve higher tolerance to temperature when they can simply produce heat-resistant spores? The presence of the same two *Bacillus* lineages in two contrasting environments, temperate and hot, provided the opportunity to evaluate the effect of evolutionary history on phenotypic plasticity as a response to temperature selection. A hot-spring constitutes selective pressure at what appears to be the edge of surviving temperature for mesophilic strains. Reaction norms, as a property of individual genotypes, allowed us to explore in a bacterial model the extent of phenotypic plasticity as the result of environmental history and also the possible genetic constraints in two lineages.

There are reports of convergent evolution in bacteria when subject to experimental evolution (Tenaillon et al., 2012). If bacteria isolated from the same environment responded in the same way to environmental challenges, despite differences in evolutionary history, this would suggest that prolonged evolution under stable conditions could lead to homogenous strategies to face environmental challenges. In our study, the evaluated species Bs and Bc lineages exhibited only intraspecific similarities rather than convergence patterns among the strains sharing a common selection regime (T or H). This suggests that deep evolutionary history of the individuals had set the genetic frame that determined their response to temperature, limiting their phenotypic plasticity. This agrees with observations that plasticity to temperature can be regarded as a species trait (Martiny et al., 2015).

Bs and Bc exhibited differences in their plasticity. The Bc lineage, with characteristic norms of reaction, showed an abrupt drop in growth after 42 °C. This behaviour has been called striking asymmetry and has been observed for reaction norms to temperature of many organisms, as performance increases and reaches an “optimal” level and then rapidly decreases near the lethal temperature (Huey & Kingsolver, 1993). In contrast, the strains belonging to the Bs lineage exhibited a broader curve of tolerance and the strains from the H environment extended their tolerance to 47 °C, ten degrees above their optimal of 37 °C. The phenotypic plasticity of Bs seems to be superior in the isolates evaluated, including the laboratory strain PY79. It has been reported that Bs strain 168 can grow up to 52 °C (Holtmann & Bremer, 2004). It is intriguing that the laboratory strain exhibits higher phenotypic plasticity to temperatures it has probably not experienced, while Bc from the hot-spring did not become more tolerant to temperatures it has experienced possibly for years. We hypothesize that the hot springs have harbored microbial mats for a long time and that their associated bacteria have had the opportunity to evolve tolerance to higher temperatures.

Another distinction between the lineages is the noticeable difference in growth and maximum growth rate (within their optimal range of temperature tolerance), with Bc strains exhibiting a faster growth rate than Bs. This also seems to be a lineage trait that did not change in either of the clades as long as it was evaluated within their thermal niche (Fig. 4A). We had expected a decrease in the doubling time of the Bc strains, from the hot-spring as a possible trade-off of the ability to sustain growth at a higher temperature. This was not observed even at 44 °C. Its ecological strategy seems to be shifted towards faster growth, maybe to compensate that it can’t sustain growth at a higher temperature. This suggests that Bc is a specialist, with an r strategy, while Bs is a generalist, given the wide breadth of its thermal niche. These characteristics could have important implications when being part of a microbial community, particularly in constraining environments (Székely & Langenheder, 2014).

Our results suggest that sporulation is a form of plasticity that limits evolution. Since the Bacillus can sporulate, their thermal niche has to be defined for the vegetative and for the full developmental program leading to spore formation. For the *Bacillus* spp. (and other microorganisms), sporulation is the ultimate survival strategy allowing them to resist harsh environmental conditions (temperatures of 70 to 80 °C) for prolonged periods (Huang & Hull, 2017). Although the *Bacillus* recovered from environment H could tolerate higher temperature in a vegetative stage than those from environment T, their optimal temperature for growth is still around 37 °C and, surprisingly, they don’t grow above 50 °C and in the vegetative phase they lose viability. For the hot spring strains, phenotypic plasticity falls short at temperatures above 44 °C to 47 °C. With a limited thermal maximum for vegetative growth, these *Bacillus* probably survive in the hot springs as spores. If, as it has been suggested, under conditions in which phenotypic plasticity is favored, genetic variation can be limited (Murren et al., 2015), in this case sporulation could limit the selection of heat tolerance in the vegetative phase.

Our data showed that strains from environment H, as expected, were able to tolerate higher temperature for growth. This is consistent with data from experimental evolution studies using temperature as a selective environment and with data of bacterial isolates from natural environments. Experimental evolution work has been done mainly in *E. coli*. Populations evolved increased competitive fitness in the thermal regime that they experienced during the experiment (Mongold, Bennett & Lenski, 1996; Bennett, Lenski & Mittler, 1992). The results from Bennett, Lenski & Mittler (1992) showed that *E. coli* strains evolved at 42 °C, can shift their tolerance towards higher temperature. Regarding bacteria from natural settings, Bronikowski et al. Bronikowski, Bennett & Lenski (2001) did not observe variation in growth profiles for *Salmonella* or *E. coli* (comparisons within groups) isolated from turtle populations (undergoing natural changes in season temperatures) and from squirrels. Notwithstanding the lack of overlap between temperature ranges of different seasons, the breadth of all isolated strains were similar no matter what host they came from. On the other hand, the work of Sikorski and Nevo (50) showed increased tolerance to temperature among *Bacillus simplex* species isolated from a southern hill, that received more solar radiation and is consequently warmer and dryer, compared to strains from the northern hill. They also observed that the strains more tolerant (*B. simplex*) to temperature did not have a reduced capacity to grow at a lower temperature (Sikorski & Nevo, 2007).

Can mesophilic *Bacillus* strains being exposed to strong selection at the limit of their temperature tolerance evolve thermophilic features or have they reached their temperature tolerance limit? Given their importance in food safety, several works have evaluated temperature tolerance in the *B. cereus sensu lato*. All in all, there are no examples of thermophilic strains in this group, and our results shows that even the strains from the hot-spring are mesophilic. Only *Bacillus cytotoxicus* had been considered thermotolerant (Guinebretière et al., 2008) but now is recognized as a novel species “*B. cytotoxicus*” having a clearly distinguishing moderate thermotolerant phenotype (Guinebretière et al., 2013) and being genetically distant from more than 200 *B. cereus* examined (Liu et al., 2015).

The genetics behind the response to temperature may constrain changes in phenotypic plasticity. Our results show that the Bs and Bc lineages can be easily identified through their norm of reaction in both environments. Phenotypic plasticity seems to be a lineage trait, and each of the *Bacillus* lineages seems to possess a distinctive reaction norm to temperature and possibly a genetic architecture that limits convergence. It is possible that substantial molecular changes may be required to increase upper thermal limits for the *Bacillus* and that the observed limited tolerance to temperature reflects evolutionary constraints. Environmentally induced plastic phenotypes are thought to be controlled by gene regulatory networks (Schlichting & Pigliucci, 1993). The heat shock response was the first regulatory system discovered and is considered one of the fundamental systems concerning general stress (Neidhardt & VanBogelen, 1987). Bacteria and lower eukaryotes share conserved families of chaperones and maybe also conserve the complexity of thermal systems such as chaperone networks (Richter, Haslbeck & Buchner, 2010). As a system, the network may be slower to evolve. This may be the reason for the resistance to change in genetic lineages and could explain the correlation in plasticity to temperature as a function of the organisms’ genetics, not of the environment.

In summary, phenotypic plasticity of temperature tolerance (thermal acclimation) is considered an important component of the evolutionary response to variable temperatures and specifically as a relevant response to climate change (Hoffmann, Chown & Clusella-Trullas, 2013). Understanding how organisms respond and adapt to novel environments is critical to our efforts to conserve biodiversity and maintain ecosystem function. The limited potential observed for hot-spring strains to change their thermal limits in a vegetative stage should be a strong warning particularly within the context of an average predicted temperature increase of 2–4 °C for mid-latitude populations over the next few decades. It would be interesting to test the hypothesis of the genetic constraints on thermal tolerance by subjecting the *Bacillus* strains from the temperate environment to experimental evolution to find their thermal boundaries, and to also explore thermal plasticity in natural isolates in non-sporulating bacteria.

## Conclusion

Despite sharing the same environment (hot or temperate), the evaluated *Bacillus* strains from the *Bc* and *Bs* lineages do not converge in their norms of reaction to temperature. Deep evolutionary differences between lineages define the genetic possibilities of plasticity to temperature, such that the norms of reaction to temperature can be considered a strong lineage signature for the Bc and Bs strains analyzed. The thermal niche of the *Bacillus* from the hot-springs was observed to be shifted only a few degrees toward more tolerance to temperature, possibly due to genetic architecture constraint. Sporulation allows the hot-spring *Bacillus* strains to exceed what would be their temperature tolerance limit in the vegetative stage, and although the spore state allows their survival, it may reduce opportunities to evolve higher tolerance. Therefore, we suggest that the life cycle of the *Bacillus* from the hot springs may be completed in two environments, with the germination and growth phases occurring only at moderate temperatures. The reduced phenotypic plasticity exhibited by these bacterial lineages should be a warning for the limited capability, even of bacteria, to adjust to climate change.

## Supplemental Information

10.7717/peerj.11734/supp-1 Supplemental Information 1 Supplementary Tables

10.7717/peerj.11734/supp-2 Supplemental Information 2 Growth of *Bacillus* f** rom a hot spring (Michoacán) and a temperate lagoon (Churince, Cuatro Cienegas) at different temperatures Dilutions of strains were plated out on semisolid Marine Medium and incubated for 24 h at 37, 44, 50 and 55 °C. Only strains from*B. subtilis sensu lato* can grow at 50 or 55 °C. Only plates with less than 20 colonies were chosen to measure the size of colonies represented in Fig. 2.

10.7717/peerj.11734/supp-3 Supplemental Information 3 Phylogenetic analysis based on the 16S rRNA gene of *Bacillus* strains from our collection from the hot-springs and the temperate lagoon of Churince A maximum-likelihood phylogenetic analysis was done based on 16S RNA gene to select strains that belonged to either the *Bacillus cereus sensu lato* (lineage B) or the *Bacillus subtilis sensu lato* (lineage A).

## Additional Information and Declarations

### DNA Deposition

The following information was supplied regarding the deposition of DNA sequences: The 16S sequences are available at NCBI: Bc_370a MK850162.1; Bc_102 MK880370; Bc_111b MK850167.1; Bc_28 MK850171.1; Bc_11 MK850156.1; Bc_51 MK850144.1) The six strains from the *B. subtilis* lineage are available at NCBI: Bs_21 MK850173.1; Bs_90 MK850153.1; Bs_427 MK850159.1; PY79 MK850170.1; Bs_2 MN180832; Bs_30 MK850157.1: Bs_45 MK850154.1.

### Data Availability

The following information was supplied regarding data availability: Raw data are available as a Supplemental File.
