# Supplementary figures and images for: Phenotypic plasticity and evolution of thermal tolerance in bacteria from temperate and hot spring environments
Source: PeerJ. 2021 Jul 23;9:e11734. doi: 10.7717/peerj.11734 (PMC8312496; doi:10.7717/peerj.11734)

*Bacillus cereus*

strain 37°C 44°C

Temperate lagoon

370a

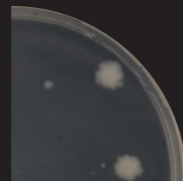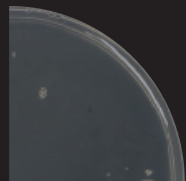

102

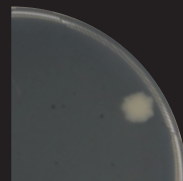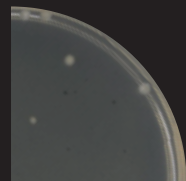

111b

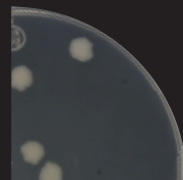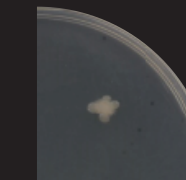

Hot springs

11

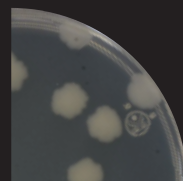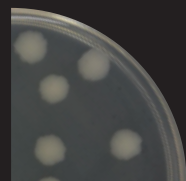

28

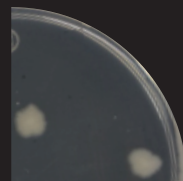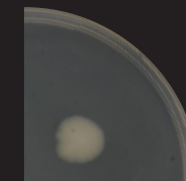

51

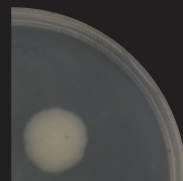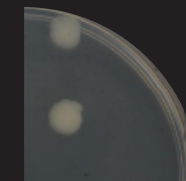

*Bacillus subtilis*

strain 37°C 44°C 50°C 55°C

21

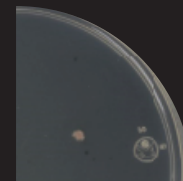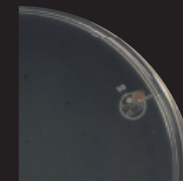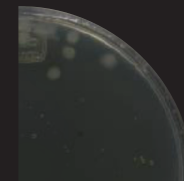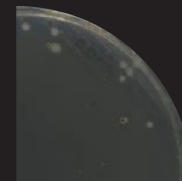

90

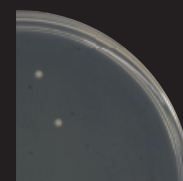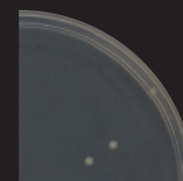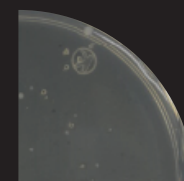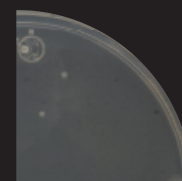

427

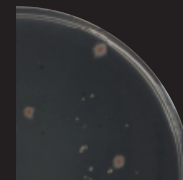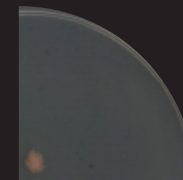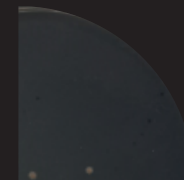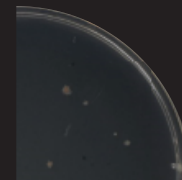

PY79

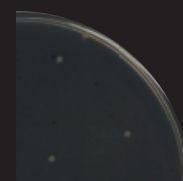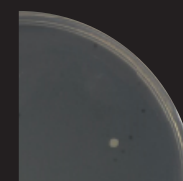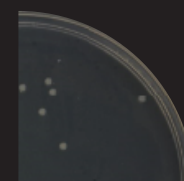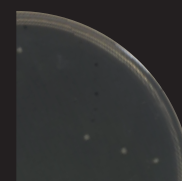

2

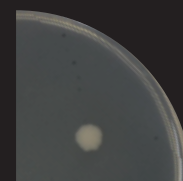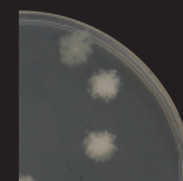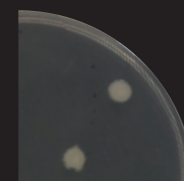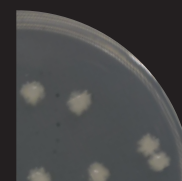

30

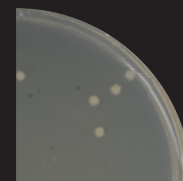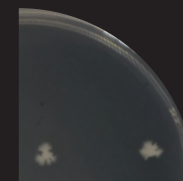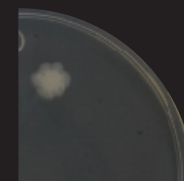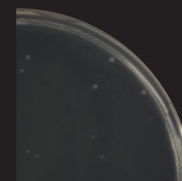

45

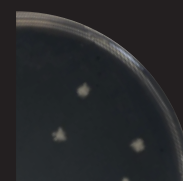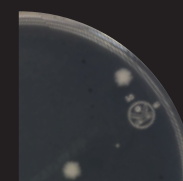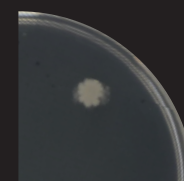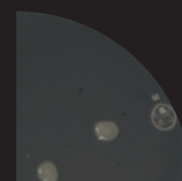

Supplement: Supplemental Information 2 — Dilutions of strains were plated out on semisolid Marine Medium and incubated for 24 h at 37, 44, 50 and 55 °C. Only strains fromB. subtilis sensu lato can grow at 50 or 55 °C. Only plates with less than 20 colonies were chosen to measure the size of colonies represented in Fig. 2. [file peerj-09-11734-s002.pdf]

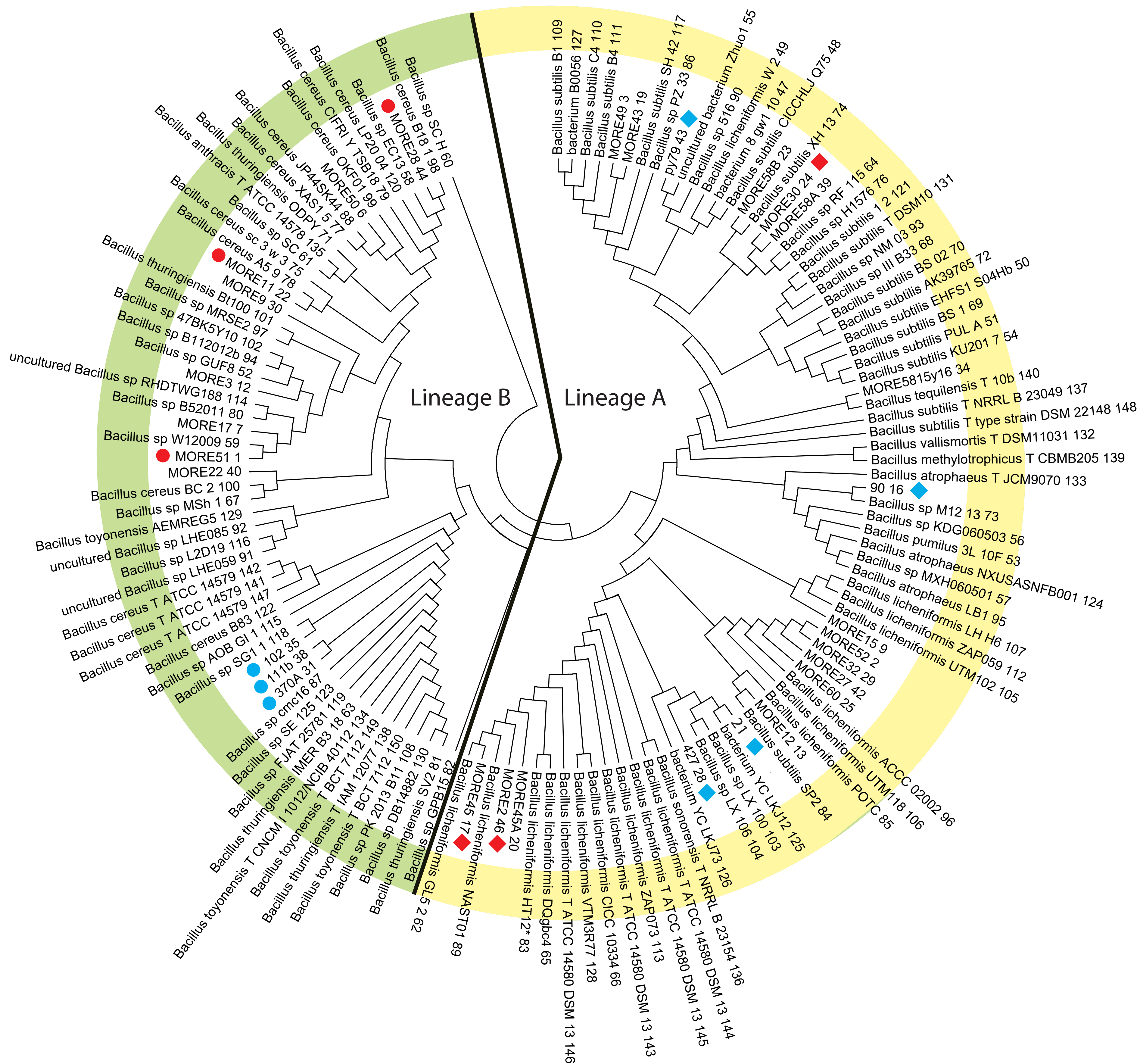

Supplement: Supplemental Information 3 — A maximum-likelihood phylogenetic analysis was done based on 16S RNA gene to select strains that belonged to either the Bacillus cereus sensu lato (lineage B) or the Bacillus subtilis sensu lato (lineage A). [file peerj-09-11734-s003.pdf]
